# Optic Nerve Sheath Diameter: Correlation With Intra-Ventricular Intracranial Measurements in Predicting Dysfunctional Intracranial Compliance

**DOI:** 10.7759/cureus.13008

**Published:** 2021-01-30

**Authors:** Seelora Sahu, Nidhi Panda, Amlan Swain, Preethy Mathew, Navneet Singla, Sunil Gupta, Kiran Jangra, Avanish Bhardwaj, Hemant Bhagat

**Affiliations:** 1 Anaesthesiology, Tata Main Hospital, Jamshedpur, IND; 2 Anaesthesia and Intensive Care, Postgraduate Institute of Medical Education and Research, Chandigarh, IND; 3 Neurosurgery, Postgraduate Institute of Medical Education and Research, Chandigarh, IND; 4 Anaesthesiology and Critical Care, Command Hospital Airforce Bangalore, Bengaluru, IND

**Keywords:** intracranial pressure, ultrasonography, optic nerve sheath diameter

## Abstract

Background

Ultrasonographic (USG) measurement of optic nerve sheath diameter (ONSD) has been proposed as a non-invasive, bedside method to detect raised intracranial pressure (ICP) in various clinical settings. We aimed to correlate the ONSD obtained from ultrasonography with the gold standard, intraventricular ICP, and to find out the cut-off point which predicts ICP accurately at different levels.

Methodology

A prospective double-blind study was carried out by performing ocular ultrasounds in 30 adult patients with features of intracranial hypertension. The ONSD was measured by USG intraoperatively along with direct intraventricular pressure measurement. The ONSD was compared with the intraventricular ICP and correlations were derived. The optimum cut-off of ONSD to predict ICP > 20 mm Hg, 25 mm Hg, 30 mm Hg, and 35 mm Hg was sought.

Results

There was a significant correlation of ONSD with ICP (r = 0.532, p = 0.002). An ONSD threshold of 5.5 mm predicted ICP > 20 mm Hg with high sensitivity (100%) and specificity (75%) (area under receiver operating characteristic [ROC] curve = 0.904, p=0.01). The optimum ONSD cut-off predicting ICP at values of 25 mm Hg, 30 mm Hg, and 35 mm Hg was 6.3 mm, 6.5 mm, and 6.7 mm, respectively.

Conclusion

Our study confirms the utility of optic nerve ultrasound in the diagnostic evaluation of patients with known or suspected intracranial hypertension. We recommend an ONSD cut-off of 5.5 mm for predicting ICP > 20 mm Hg.

## Introduction

Diagnosis and management of raised intracranial pressure (ICP) is a critical aspect of patient care in neurological and neurosurgical cases [1]. Early detection and treatment of intracranial hypertension, defined as an ICP greater than or equal to 20 mm Hg, plays a significant role in the avoidance of secondary brain injury [2-6]. If increased intracranial pressure is severe enough, herniation of portions of the brain from their normal location into other compartments over the dural membranes may occur, leading to compression of adjacent brain structures, giving rise to uncal, central, transtentorial, and downward herniation syndromes with catastrophic consequences [2-6]. 

Intraventricular measurement of intracranial pressure remains the gold standard for measurement of ICP [7]. However, its association with various complications like infection, trauma, hemorrhage, and the inability to cannulate or perform it as a rapid bedside method is a significant limitation of the invasive method [8-9]. This has led to an intense search for non-invasive methods of measuring intracranial pressure [10]. Known methods of non-invasive ICP measurement like tympanic membrane displacement techniques, transcranial doppler ultrasonography, magnetic resonance imaging, computerized tomography scans, quantitative pupillometry, and optic nerve sheath diameter measurements eliminate the aforementioned complications related to invasive methods [10]. However, they have limited accuracy and reproducibility, hence, limiting their routine and widespread use in clinical practice [10]. Recent research in this field has been extensive and there is still an ongoing search for a non-invasive method of ICP monitoring that is convenient to perform, fairly accurate, and easily reproducible.

Measurement of optic nerve sheath diameter (ONSD) has been explored as a non-invasive, bedside method, to detect intracranial hypertension. It is routinely performed by physicians, intensivists and medical personnel, especially in medical establishments caring for neurosurgical and neurological patients. ONSD measurement is based on the premise that there exists a constant communication between the subarachnoid space of the optic nerve sheath and the intracranial cavity [11], and hence, changes in the ICP can be reliably detected by changes in the diameter of optic nerve sheath [12]. Expansion of the leptomeningeal sheath of the optic nerve forms the basis of ONSD measurements and is most markedly seen at a depth of 3 mm from the posterior pole of the eyeball, the point thought to be most reflective of any changes in the intracranial pressure [12]. The cut-off value for optic nerve sheath diameter to predict intracranial hypertension ranges from 4.8 mm to 6.0 mm in previous studies [13]. We endeavored to correlate the ONSD measured by ultrasonography with the opening cerebrospinal fluid (CSF) pressures measured by an intraventricular catheter reflecting the ICP values, in patients with features suggestive of raised intracranial pressure.

## Materials and methods

A prospective, observational study was conducted on 30 adult patients at the Neurosurgery Operation Theatre, Main Operation Theatre Complex, Nehru Hospital, Postgraduate Institute of Medical Education and Research, Chandigarh, India, after obtaining Institutional Ethical Committee approval. Thirty patients, in the age group of 18-65 years, undergoing surgical procedures for raised intracranial pressure, were included in the study and informed written consent was obtained from each patient or their nearest relation. Patients having documented or reported or clinical evidence of orbital pathologies, orbital injuries, disease affecting the optic nerve, as well as mechanical complications such as the inability to cannulate lateral ventricle and trauma and bleeding during ventricular cannulation, were excluded from the study. None of the patients had an intraventricular catheter in situ before the study.

The ultrasonographic ONSD values were compared with the opening CSF pressure values obtained through a catheter placed in the cerebral ventricles. These ICP values were used for deriving correlations as well as to find the optimum cut-off of ONSD (measured by ultrasonography) that could reliably predict intracranial hypertension at different levels of raised ICP (at 20, 25, 30, and 35 mm Hg).

Measurements

*ONSD Measured from Ultrasonography: *The ultrasonographic measurements were performed in the supine position using a 13-6 MHz linear probe (L25e, Sonosite MicroMaxx, SonoSite Inc., Bothell, WA, USA), placed over the closed eyelid on the upper temporal side, in accordance with previous studies employing the same modality [12]. The distance between the external borders of the hyperechoic area surrounding the optic nerve, 3 mm behind the globe was measured by electronic calipers. Three scans in each eye were performed and an average of these six measurements was used for calculating the representative ONSD for a particular patient. The ONSD was measured by ultrasonography after inducting anesthesia at nearly the same time as intraventricular ICP was measured immediately after the placement of the intraventricular catheter, as described below.

*Invasive ICP Measurement: *For the intracranial pressure measurements, a ventriculostomy catheter was inserted into the lateral ventricle after dural reflection, through a parietal or frontal burr hole. Taking utmost care in preventing any loss of CSF, the stylet of the ventriculostomy catheter was removed and the catheter was connected to a pressure transducer system (package transducer Edwards; TruWave™ 3 cc/84 in (210 cm), Edwards Lifesciences, Irvine, CA, USA) and the intracranial pressure was measured with a Philips MP40 IntelliVue monitor (Philips Medical System, Best, The Netherlands).

The intraventricular pressures thus measured were recorded at one-minute intervals for three minutes. The average of these values was considered as the opening intracranial pressure of the patient as this pressure would have caused the maximum distention of the optic nerve sheath.

*Anesthesia Technique: *All the patients received anesthesia according to a standard protocol that included induction with propofol (1-2 mg/kg) titrated to loss of verbal response and trachea was intubated following neuromuscular blockade in the form of vecuronium bromide (0.1 mg/kg). Maintenance of anesthesia was accomplished with isoflurane (MAC - 0.8-1) along with nitrous oxide-oxygen mixture (60:40). Ventilation was controlled so as to achieve a partial pressure of carbon dioxide (PaCO2) in the range of 35 to 45 mm Hg. Intra-operative analgesia was maintained with intravenous fentanyl with a bolus of 2 μg/kg followed by an infusion of the same at the rate of 1 μg/kg/hr.

The anesthesiologist (Nuse Practitioner) doing the sonographic ONSD measurements was blinded to the intraventricular ICP. All the measurements were collated and analyzed by an anesthesiologist who was not involved in any of the ONSD or ICP measurements.

Data recorded included demographic variables like age, sex, weight, American Society of Anesthesiologist (ASA) status, diagnosis, duration of presenting complaints, and preoperative Glasgow Coma Scale (GCS). The hemodynamic variables (heart rate, systolic blood pressure, diastolic blood pressure, mean arterial pressure), pulse oximetry (SpO2), end-tidal carbon dioxide (EtCO2), and minimum alveolar concentration (MAC) of the anesthetic agents at the time of the ONSD and the ICP measurement.

*Statistical Analysis: *Data analysis was performed with the aid of Statistical Package for the Social Sciences software (version 20, IBM Corp., Armonk, NY, USA). Parametric values were expressed as mean ± standard deviation. The distribution of the study measurements was verified using Shapiro-Wilk test. Pearson test was used to evaluate the correlation between the ONSD measured by ultrasonography with the intraventricular ICP. Sensitivity and specificity of the optic nerve sheath diameter measurements to diagnose increased ICP was calculated at various cut-off points at 95% confidence interval; receiver operating characteristic (ROC) curve was used to calculate the area under the curve in order to find the optimal cut-off point. Statistical significance was defined at p < 0.05.

## Results

The study was carried out in 30 patients with features of raised ICP. The general characteristics of patients are summarized in Table 1.

**Table 1:** Demographic parameters Values expressed as mean ± standard deviation, n (percentage) GCS = Glasgow Coma Score; ICP = intracranial pressure

Parameter		Mean ± standard deviation N (%)
Age ( in years)		32.73 ± 13.13
Weight ( in kg)		59.33 ± 10.35
Sex ( male: female)		13:17
GCS		14.93 ± 0.25
Duration of presenting symptoms		59.93 ± 56.17
Presenting symptom (number of patients)	Headache	24 (80)
	Vomiting	21(70)
	Vision disturbance	2(7)
	Neurological deficit	3(10)
Aetiology of raised ICP (number of patients)	Obstructive due to mass lesion	22(73)
	Congenital Aqueductal Stenosis	3(10)
	Aneurysm	4(13.3)
	Infectious	1(3.3)
Comorbidity/Other finding	Hypertension	2(1.3)
	Hypothyroid	1(3.3)
	Pregnancy	1(3.3)

There were 26 patients with an ICP of > 20 mm Hg and four patients with an ICP recording of < 20 mm Hg. Fifteen patients had an ICP > 25 mm Hg, five had an ICP > 30 mm Hg and one patient had an ICP > 35 mm Hg. There were 22(73%) patients diagnosed with obstructive hydrocephalous secondary to intracranial space-occupying lesions, three (10%) patients had congenital hydrocephalous, four (13%) patients had aneurysmal subarachnoid hemorrhage, and 1(3.3%) patient had infectious etiology. The patients presented with headache (n=24), vomiting (n = 21), neurological deficit (n = 3), and vision deficit (n =2). All patients belonged to American Society of Anesthesiology (ASA) I (n = 25) or ASA II (n = 5) physical status. Fourteen patients (46%) underwent endoscopic third ventriculostomy, 12 (40%) had ventriculoperitoneal shunt placement, and four (13%) patients with intracranial aneurysm required CSF drainage surgery. The demographic and hemodynamic parameters were comparable between the patients with intracranial hypertension (ICP > 20 mm Hg) and those without intracranial hypertension (ICP < 20 mm Hg) (Tables 2, 3).

**Table 2:** Demographic parameters between the High ICP (> 20 mm of Hg) and Low ICP (< 20 mm of Hg) groups Values expressed as mean ± standard deviation, p < 0.05 considered significant. ONSD = optic nerve sheath diameter, ICP = intracranial pressure.

	High ICP group (n = 26)	Low ICP group (n = 4)	p-value
Age (in years)	32.85 ±13.89	32.00 ± 7.87	0.907
Weight ( in kg)	59.27 ± 10.88	59.75 ± 6.94	0.933
GCS	14.92 ± 0.27	15.00 ± 0.00	0.581
Duration of symptoms ( in days)	56.92 ± 54.8	75.00 ± 74.41	0.711
Time gap between measurements	14:25 ± 3:28	13:45 ± 2:30	0.588

**Table 3:** Hemodynamic parameters between High ICP and Low ICP groups Values expressed as mean ± standard deviation, p < 0.05 considered significant ONSD = optic nerve sheath diameter; ICP = intracranial pressure; SBP = systolic blood pressure; DBP = diastolic blood pressure; MBP = mean blood pressure; SPO2 = oxygen saturation; ETCO2 = end-tidal carbon dioxide;  MAC = minimum alveolar concentration

Parameter		ICP > 20 (n = 26)	ICP < 20 (n = 4)	p-value
Baseline	Heart rate (in beats/ min)	84.04 ± 14.04	76.00 ± 10.19	0.283
	SBP (in mm of Hg)	122.46 ± 25.57	111.17 ± 12.81	0.392
	DBP (in mm of Hg)	75.08 ±13.99	64.5 ± 10.27	0.160
	MAP (in mm of Hg)	90.85 ± 12.80	84.5 ± 12.93	0.236
	SPO2 (in %)	99.00 ± 1.42	98.75 ± 2.5	0.772
During ONSD and ICP measurement	Heart rate ( in beats/ min)	67.15 ± 11.89	74.50 ± 13.57	0.267
	SBP (in mm of Hg)	103.08 ± 15.91	98.00 ± 2.16	0.535
	DBP (in mm of Hg)	59.00 ± 13.71	60.00 ± 4.54	0.888
	MAP (in mm of Hg)	75.15 ± 12.78	76.25 ± 8.34	0.870
	SPO2 (in %)	99.85 ± 0.46	99.75 ± 0.50	0.705
	EtCO2 (in mm of Hg)	29.15 ± 2.07	31.25 ± 0.50	0.057
	MAC	0.95 ± 0.08	0.93 ± 0.05	0.490

ONSD and ICP measurements

A normal distribution of variables was observed for the ONSD measured by ultrasonography (p = 0.185) and the intraventricular ICP (p = 0.277) measurements. The mean, median, and mode of ONSD measurements was 6.31 ± 0.61 mm (range 5.2 - 7.4 mm), 6.44 mm, 7.17 mm, and ICP measurement was 24.77 ± 7.11 mm Hg (range 8 - 45 mm Hg), 25.0 mm Hg, 28.0 mm Hg, respectively.

Correlations

**Figure 1:**
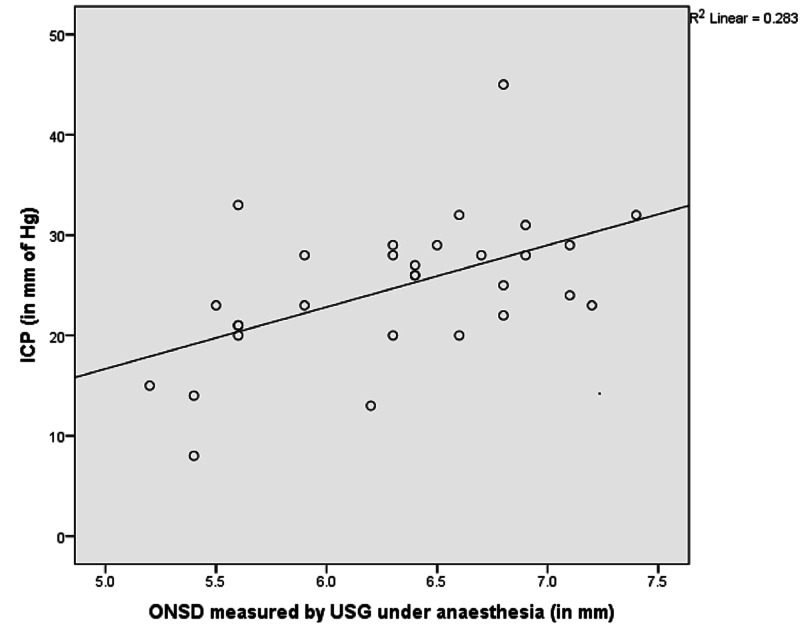
Scatter plot showing correlation between ONSD measured by ultrasonography and the ICP ONSD = optical nerve sheath diameter; ICP = intracranial pressure; USG = ultrasonography

We found a statistically significant correlation between the ONSD values and the intraventricular ICP (r = 0.532, p = 0.002) (Table 4, Figure 1).

**Table 4:** Pearson correlation between ONSD and ICP * Correlation is significant at the 0.01 level (2-tailed) ONSD = optic nerve sheath diameter; ICP = intracranial pressure

Correlations			
		ONSD	ICP(max)
ONSD	Pearson Correlation	1	0.532^*^
	Sig. (2-tailed)		0.002
	N	30	30
ICP(max)	Pearson Correlation	0.532^**^	1
	Sig. (2-tailed)	0.002	
	N	30	30

Optimum cut-offs

**Figure 2:**
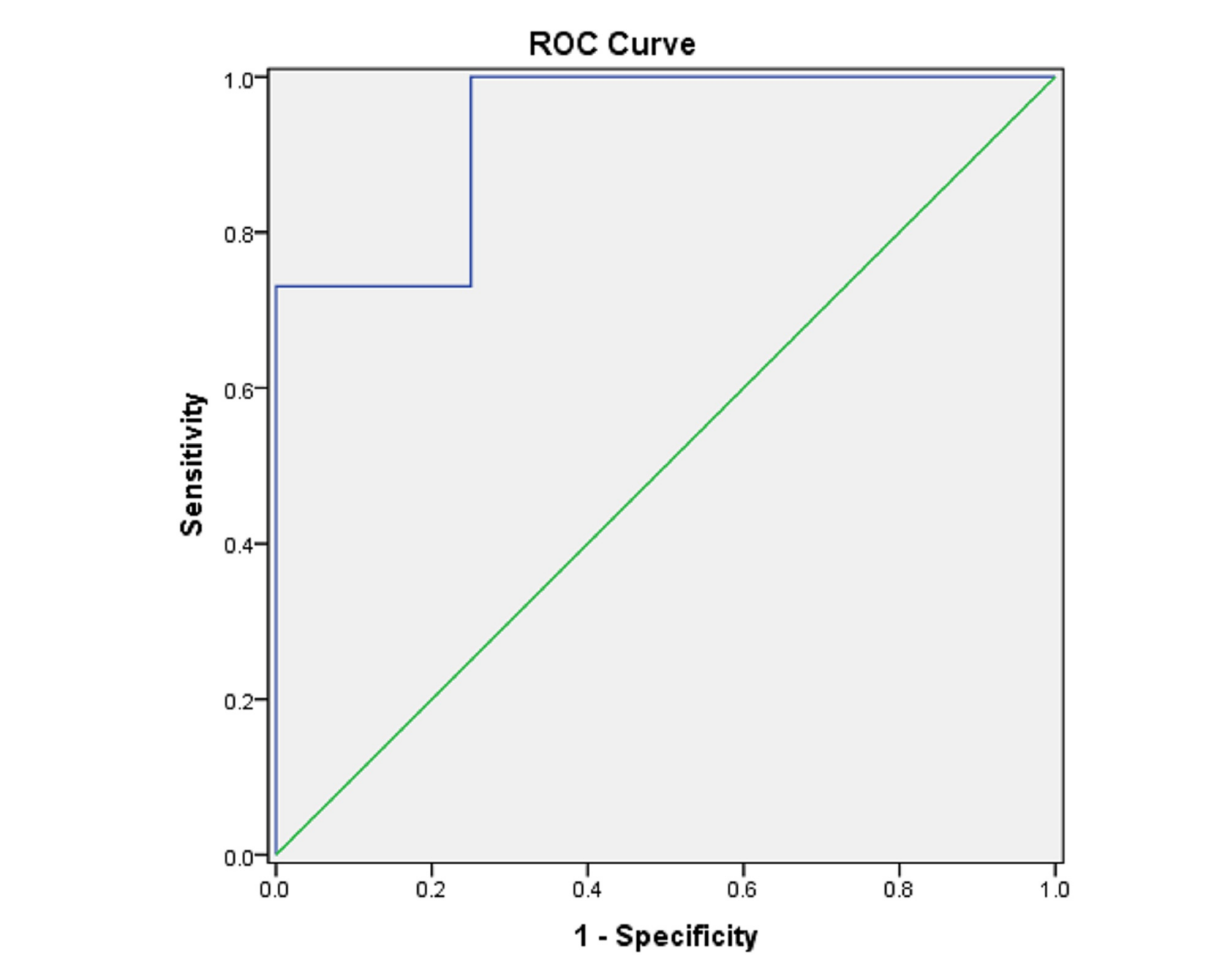
ROC curve for ICP 20 mm Hg ROC = receiver operating characteristic; ICP: intracranial pressure

The higher ONSD values suitably predicted ICP ≥ 20 mm of Hg. The area under ROC was 0.933 (p = 0.00000), which was significant with a 95% confidence interval (0.805-1.000) (Table 5, Figure 2).

**Table 5:** Area under the curve for ICP 20 mm hg ^a ^Under the nonparametric assumption ^b ^Null hypothesis: true area = 0.5 ONSD = optic nerve sheath diameter, ICP = intracranial pressure

Test Result Variable(s): ONSD				
Area	Std. Error^a^	Asymptotic Sig.^b^	Asymptotic 95% Confidence Interval	
			Lower Bound	Upper Bound
0.933	0.065	0.006	0.805	1.000

An ICP ≥ 20 mm of Hg was predicted by a cut off of 5.5 mm with a sensitivity of 100%, specificity of 75%, a positive likelihood ratio of 4 and a positive predictive value of 95%.

Similarly, the optimum ONSD cut-off predicting ICP at values of 25 mm Hg, 30 mm Hg, and 35 mm Hg was 6.3 mm, 6.5 mm, and 6.7 mm respectively (Table 6).

**Table 6:** Optimum ONSD cut-off to predict ICP Data expressed as value, area under curve, percentage (%), p-value (p < 0.05 considered significant) *statistically significant values. ONSD = optic nerve sheath diameter; ICP = intracranial pressure; ROC = receiver operating characteristic

ICP cut-off	ONSD (in mm)	Area under ROC curve	Sensitivity (%)	Specificity (%)	p value
20 mm Hg	5.5	0.933	100	75	0.006*
25 mm Hg	6.3	0.810	88.2	70	0.004*
30 mm Hg	6.5	0.716	80	68	0.133
35 mm Hg	6.7	0.759	100	73	0.386

## Discussion

Our study establishes the utility of trans-orbital sonographic measurement of ONSD for detecting intracranial hypertension in 30 adult patients with clinical features of raised ICP.

Among the 30 patients in our study group, there were more patients (n = 26) who had an ICP more than or equal to 20 mm Hg, which can be attributed to the fact that all patients in the study group presented with clinical features of raised ICP. All the demographic, anesthetic, and hemodynamic parameters were comparable between the patients with high ICP (more than 20 mm Hg, n = 26) and low ICP (less than 20 mm Hg, n = 4), thus eliminating the role of these factors as confounding variables that might affect the results. The fact that controlled ventilation resulted in PaCO2 values in the normocapnic range eliminates the role of carbon dioxide in blood as a confounding variable affecting ICP.

In previous studies, the ultrasonographic measurement of ONSD has revealed an excellent intra-observer and interobserver reproducibility (0.25 to 0.3 mm) in persons with normal ICP [14-16]. It has also demonstrated a rapid response to any changes in ICP [12]. The correlation of ONSD with intracranial pressure has been previously validated in a few studies. Ultrasonographic measurement of ONSD has been correlated with CT and MRI findings suggestive of raised ICP, intracranial pressure measured by intraparenchymal probes, bolts, external ventricular drainage (EVD) catheters and CSF pressures measured by lumbar puncture [3, 14-16, 17-19]. However, as of now, there has been no study correlating ONSD measured by ultrasonography with the opening ICP measured by an intraventricular catheter, which logically and logistically remains the gold standard for ICP measurements.

We compared the ultrasonographic ONSD with the opening ICP measured invasively with an intraventricular catheter in order to assess the accuracy of ONSD to predict raised ICP. The results of our study suggest that an ONSD cut off of 5.5 mm is a strong predictor of ICP of > 20 mm Hg. In a similar study Kimberley et al. reported an ONSD cut off of 5.0 mm to predict an ICP > 20 mm Hg [17]. However, they used an EVD in situ for the ICP measurements, where, as a consequence of ongoing drainage of CSF, it can be hypothesized that the measured ICP values would not accurately reflect intracranial hypertension of the highest severity, thereby producing a lower ONSD cut-off for prediction of intracranial hypertension. The study by Soldatos et al., which reported a cut-off value of 5.7 mm, used Camino intraparenchymal probes, which are themselves associated with complications like drift and also reflect the compartmental pressure instead of global ICP [19, 20]. Moretti et al. demonstrated an ONSD threshold of 5.2 mm as a predictor of intracranial hypertension in patients where ICP monitoring was done with EVD or intraparenchymal probes [16]. Bäuerle et al. compared the ultrasonographic ONSD with the opening CSF pressures measured by lumbar puncture and concluded that the best cut-off value of ONSD for detecting raised ICP was 5.8 mm [21]. Similarly, Jeon et al. advocated an ONSD cut off of 5.6 mm using EVD and Robba et al. proposed an ONSD of 5.85 mm using intraparenchymal probes and EVD [22,23]. More recently, a meta-analysis of these recent studies by Robba et al. has validated the potential usefulness of ONSD in predicting ICP noninvasively [24]. The fact that we used opening intraventricular pressure makes the ICP values more accurate and precise representation of intracranial compliance especially in comparison to the aforementioned studies using EVD, intraparenchymal probes, and lumbar CSF pressure [25].

The inherent strength of our study is that it predicts an ONSD value of 5.5 mm based on direct intraventricular measurements of opening CSF pressure, without any loss of CSF, making it a more accurate method of validation for sonographic ONSD measurements for raised ICP. Our study revealed a significant correlation between ultrasonographic ONSD and invasive ICP (r = 0.532, p = 0.002). Studies such as those done by Geeraerts et al. [15], Bäuerle et al. [21], Rajajee et al. [18], and Kimberly et al. [17] have shown a correlation between ultrasonographic ONSD and invasive ICP in the range of 0.46 and 0.74 (p = 0.0001 to p = 0.02). These studies have demonstrated a sensitivity in the range of 74% to 96% and a specificity of 74% to 100% for an ONSD cut off in the range of 4.8-5.9 mm [13, 18-20]. Our study demonstrated a sensitivity of 100% and specificity of 75%.

In addition, we tried to find the optimum cut-off values of ONSD which could predict ICP at different levels other than the conventional 20 mm Hg. The ONSD values predicting ICP at 25, 30, and 35 mm Hg were was 6.3 mm, 6.5 mm, and 6.7 mm, respectively. Previous studies have shown that the effect of increasing ICP on the ONSD seemed to plateau at an ONSD of just over 7 mm [26]. The ONSD cut-offs that we found out are well within this limit of 7 mm and could be predictive of the actual ICP. Although the ONSD values for predicting ICP at 20 mm Hg and 25 mm Hg were statistically significant, it was not the same for ICP of 30 mm Hg and 35 mm Hg. This can be attributed to the fact that we had a lesser number of readings of ICP more than 30 mm Hg (n = 5) and 35 mm Hg (n = 1). A larger sample size with more readings of ICP above 30 mm Hg might give more accurate information about the predictability of ONSD at such high ICP. The aforementioned observations warrant further research and the possible use of sonographic ONSD measurement to check for any suspected rise in ICP intra-operatively as well.

All the patients in our study received fentanyl for intraoperative analgesia and it has been proved to be similar to other opioid analgesics as regards its effect on ICP, cerebral blood flow (CBF), and brain tissue oxygenation [27]. All the cases in our study were done with isoflurane as the anesthetic agent in oxygen and nitrous oxide (40%) maintaining a combined MAC of 0.8-1. Studies with isoflurane have reported that its effects on the rate of CSF absorption are dose-related. The rate of CSF absorption was normal at 0.6% (end-expired) isoflurane, increased at 1.1%, and decreased at 1.7% and 2.2% [28]. Nitrous oxide (66%) is reported to produce no change in the rate of CSF absorption or the rate of its formation [28]. Hence, we can safely assume that the administration of anesthesia in our study population has had minimal to no effects on CSF physiology and consequently on resultant ICP thus not affecting our study results.

Limitations

The patients in our study had variable etiology for raised ICP and different duration of presentation, which can have a bearing on the extent of dilatation of the optic nerve sheath. We have not sought any correlation of the side of lesion with the ONSD of the same side. This can be an area of further study. A further limitation of our study is that we do not have any controls in our study who have normal ICP. We have however considered previous studies in patients with normal ICP as the reference for normal ONSD measurements, and in turn concentrated on patients with high ICP as there is a dearth of studies comparing these parameters (ONSD by ultrasonography and intraventricular ICP) in patients with intracranial hypertension.

There have been some concerns regarding the use of B scan ultrasounds for measurement of ONSD due to lack in sensitivity setting because of the blooming effect. This can lead to variations in the ONSD measurements and can be mitigated by use of A scans in future studies [29,30].

Since there were a small number of patients having ICP at higher levels (>30 mm of Hg), we could not demonstrate appropriate ONSD values for predicting high ICP above this range, with a sensitivity and specificity that can be clinically useful. Further studies with more number of patients with ICP in higher range may be useful in this respect.

## Conclusions

The results of our study amply demonstrate that an ONSD cut off of 5.5 mm of the optic nerve sheath diameter measured by ultrasound predicts an ICP of 20 mm Hg or more with reliable accuracy. Hence it would be prudent to conclude that the optic nerve sheath diameter measured by trans-orbital ultrasonography shows a good level of diagnostic accuracy to detect intracranial hypertension in adult patients with clinical features suggestive of raised intracranial pressure.

## References

[1] Juul N, Morris GF, Marshall SB, Marshall LF (2000). Intracranial hypertension and cerebral perfusion pressure: influence on neurological deterioration and outcome in severe head injury. The Executive Committee of the International Selfotel Trial. J Neurosurg.

[2] Schwab S, Aschoff A, Spranger M, Albert F, Hacke W (1996). The value of intracranial pressure monitoring in acute hemispheric stroke. Neurology.

[3] Gjerris F, Brennum J (2004). The cerebrospinal fluid, intracranial pressure and herniation of the brain. Clinical Neurology and Neurosurgery.

[4] Bratton S L, Chesnut R M, Ghajar J (2007). VI. Indications for intracranial pressure monitoring. J Neurotrauma.

[5] Ghajar J (2000). Traumatic brain injury. Lancet.

[6] (2006). Brain Trauma Foundation. http://braintrauma.org/.

[7] Rosenberg JB, Shiloh AL, Savel RH, Eisen LA (2011). Non-invasive methods of estimating intracranial pressure. Neurocrit Care.

[8] Binz DD, Toussaint III LG, Friedman JA (2009). Hemorrhagic complications of ventriculostomy placement: a meta-analysis. Neurocrit Care.

[9] Gardner PA, Engh J, Atteberry D, Moossy JJ (2009). Hemorrhage rates after external ventricular drain placement: clinical article. J Neurosurg.

[10] Raboel PH, Bartek J Jr, Andresen M, Bellander BM, Romner B (2012). Intracranial pressure monitoring: invasive versus non-invasive methods - a review. Crit Care Res Pract.

[11] Hyrech SS (1968). Pathogenesis of oedema of the optic disc. Doc Opthalmol.

[12] Hansen HC, Helmke K (1997). Validation of the optic nerve sheath response to changing cerebrospinal fluid pressure: ultrasound findings during intrathecal infusion tests. J Neurosurg.

[13] Kristiansson H, Nissborg E, Bartek J Jr, Andresen M, Reinstrup P, Romner B (2013). Measuring elevated intracranial pressure through noninvasive methods: a review of the literature. J Neurosurg Anesthesiol.

[14] Geeraerts T, Launey Y, Martin L, Pottecher J, Vigué B, Duranteau J, Benhamou D (2007). Ultrasonography of the optic nerve sheath may be useful for detecting raised intracranial pressure after severe brain injury. Intensive Care Med.

[15] Geeraerts T, Merceron S, Benhamou D, Vigué B, Duranteau J (2008). Non-invasive assessment of intracranial pressure using ocular sonography in neurocritical care patients. Intensive Care Med.

[16] Moretti R, Pizzi B, Cassini F, Vivaldi N (2009). Reliability of optic nerve ultrasound for the evaluation of patients with spontaneous intracranial hemorrhage. Neurocrit Care.

[17] Kimberly HD, Shah S, Marill K, Noble V (2008). Correlation of optic nerve sheath diameter with direct measurement of intracranial pressure. Acad Emerg Med.

[18] Rajajee V, Vanaman M, Fletcher JJ, Jacobs TL (2011). Optic nerve ultrasound for the detection of raised intracranial pressure. Neurocrit Care.

[19] Soldatos T, Karakitsos D, Chatzimichail K, Papathanasiou M, Gouliamos A, Karabiniset A (2008). Optic nerve sonography in the diagnostic evaluation of adult brain injury. Crit Care.

[20] Wolfla CE, Luerssen TG, Bowman RM, Putty TK (1996). Brain tissue pressure gradients created by expanding frontal epidural mass lesion. J Neurosurg.

[21] Bäuerle J1, Nedelmann M (2011 Nov). Sonographic assessment of the optic nerve sheath in idiopathic intracranial hypertension. J Neurol.

[22] Jeon JP, Lee SU, Kim SE Correlation of optic nerve sheath diameter with directly measured intracranial pressure in Korean adults using bedside ultrasonography. PLoS One.

[23] Robba C, Cardim D, Tajsic T Ultrasound non-invasive measurement of intracranial pressure in neurointensive care: A prospective observational study. PLoS Med.

[24] Robba C, Santori G, Czosnyka M (2018 Aug). Optic nerve sheath diameter measured sonographically as non-invasive estimator of intracranial pressure: a systematic review and meta-analysis. Intensive Care Med.

[25] Doherty CM, Forbes RB (2014). Diagnostic lumbar puncture. Ulster Med J.

[26] Albeck MJ, Børgesen SE, Gjerris F, Schmidt JF, Sorensen PS (1991). Intracranial pressure and cerebrospinal fluid outflow conductance in healthy subjects. J Neurosurg.

[27] de Nadal M, MunarF MunarF, Poca MA, Sahuquillo J, Garnacho A, Rossello J (2000). Cerebral hemodynamic effects of morphine and fentanyl in patients with severe head injury. Anaesthesiology.

[28] Artru AA (1989). Concentration-related changes in the rate of CSF formation and resistance to reabsorption of CSF during enflurane and isoflurane anesthesia in dogs receiving nitrous oxide. J Neurosurg Anesthesiol.

[29] Rosa N, Vitiello L, De Bernardo M (2019). Optic nerve sheath diameter measurement in hypoxic ischaemic brain injury after cardiac arrest. Resuscitation.

[30] De Bernardo M, Vitiello L, Rosa N (2019). Ocular ultrasound assessment to estimate the risk of increased intracranial pressure after traumatic brain injury in prehospital setting. Prehosp Emerg Care.

